# DEVELOPMENT AND VALIDATION OF THE TESTAMENTARY CAPACITY MEASURE AMONG OLDER ADULTS AND PATIENTS WITH DEMENTIA

**DOI:** 10.1093/geroni/igz038.2594

**Published:** 2019-11-08

**Authors:** Christopher M Nguyen, Cady Block, John Linck, Natalie Denburg

**Affiliations:** 1 The Ohio State University Wexner Medical Center, Columbus, Ohio, United States; 2 The Ohio State Wexner Medical Center, Columbus, Ohio, United States; 3 University of Oklahoma Health Sciences Center, Oklahoma City, Oklahoma, United States; 4 University of Iowa Hospitals and Clinics, Iowa City, Iowa, United States

## Abstract

As the growth of the population aged 65 and older is projected to be one of the most substantial demographic trends in history, geriatricians and other professionals working with older adults will be regularly consulted for opinion regarding an individual’s testamentary capacity (Brenkel et al., 2018). For individuals with severe cognitive and psychiatric impairment, the reduced capacity to make decision is evident. However, testamentary capacity among individuals with mild or moderate cognitive impairment has been mixed: some patients with mild cognitive impairment may be incapable of will making, while other patients with moderate cognitive impairment may have testamentary capacity (Spar & Garb, 1992). While several measures of testamentary capacity have been developed, these instruments are either not available for clinicians or lacks the sophistication of a comprehensive assessment (Marson, Huthwaite, & Herbert, 2004; Papageorgiou et al., 2018). With increasingly complex modern family structures (e.g., multiple marriages and stepchildren from these relationships), together with a projected largest transfer of wealth in human history about to occur in the next 30 years (Havens and Schervish, 2003), more standardized assessment procedures for testamentary capacity will be valuable for clinicians working with geriatric populations. In this study, healthy community-dwelling older adults and patients with Major Neurocognitive Disorder due to Alzheimer’s disease were recruited to participate in a validation study of a proposed testamentary capacity measure. Preliminary findings and implications are discussed.

